# Cohabitation—relationships of corynebacteria and staphylococci on human skin

**DOI:** 10.1007/s12223-014-0326-2

**Published:** 2014-06-01

**Authors:** Anna Kwaszewska, Maria Sobiś-Glinkowska, Eligia M. Szewczyk

**Affiliations:** Department of Pharmaceutical Microbiology and Microbiological Diagnostics, Medical University of Łódź, Pomorska 137, Łódź, 90-235 Poland

## Abstract

Skin microbiome main cultivable aerobes in human are coagulase-negative staphylococci and lipophilic corynebacteria. *Staphylococcus* strains (155) belonging to 10 species and 105 strains of *Corynebacterium* belonging to nine species from the skin swabs of healthy male volunteers were investigated to determine their enzymatic activity to main metabolic substrates: carbohydrates, proteins, lipids, and response to factors present on the skin such as osmotic pressure, pH, and organic acids. The results showed that lipophilic corynebacteria have different capacity for adaptation on the skin than staphylococci. Most of *Corynebacterium* spp. expressed lack of proteinase, phospholipase, and saccharolytic enzymes activity. Corynebacteria were also more sensitive than *Staphylococcus* spp. to antimicrobial agents existing on human skin, especially to low pH. These characters can explain domination of *Staphylococcus* genera on healthy human skin. It can be suggested that within these two bacterial genus, there exists conceivable cooperation and reciprocal protection which results in their quantitative ratio. Such behavior must be considered as crucial for the stability of the population on healthy skin.

## Introduction

Interactions of commensal microbes and host undergo extensive research in many laboratories nowadays (Arumugam et al. 2011). Examination of metagenome, including characteristics of genome, mRNA, proteins, and products of metabolism gives a novel trend in the research of human physiological flora which has been recently called microbiome (Turnbaugh et al. 2007). Natural flora plays an important role in a process of preventing colonization of the skin by pathogenic organisms known as colonization resistance of the skin. This consists of a number of mechanisms such as non-specific immune system stimulation, production of inhibitory substances by bacteria, competitive inhibition on binding sites and competition for nutrients, and acidification by organic acids or release of fatty acids (Fredricks 2001). These mechanisms also have influence on maintaining diverse resident populations characteristic for each person. The study of phenotypic features of the skin microflora can lead to a better understanding of these mechanisms. Among aerobic bacteria *Staphylococcus* and *Corynebacterium* genera are the major residents on healthy human skin. In this paper, features of *Staphylococcus* and lipophilic *Corynebacterium* strains which determine their coexistence on healthy human skin are presented.

## Materials and methods

### Bacterial strains

Strains of *Staphylococcus* (155) belonging to species *Staphylococcus epidermidis*, *Staphylococcus capitis* subsp*. capitis*, *Staphylococcus capitis* subsp*. ureolyticus*, *Staphylococcus lugdunensis*, *Staphylococcus haemolyticus*, *Staphylococcus warneri*, *Staphylococcus hominis*, *Staphylococcus caprae*, *Staphylococcus auricularis*, *Staphylococcus xylosus*, and *Staphylococcus simulans* identified according to Freney et al. (1999) and our previous study (Kaźmierczak and Szewczyk 2004) and 105 strains of *Corynebacterium* belonging to species *Corynebacterium jeikeium*, *Corynebacterium tuberculostearicum*, *Corynebacterium afermentans* subsp*. lipophilum* and *Corynebacterium urealyticum*, *Corynebacterium accolens*, *Corynebacterium diphtheriae* var. *intermedius*, *Corynebacterium kroppenstedtii*, *Corynebacterium macginleyi*, and *Corynebacterium pseudogenitalium* isolated from the skin of the forehead and the back of five healthy men were investigated. Corynebacteria were identified using numerous biological and biochemical characters (colony and cell morphology; lipophilism; nitrate reduction; CAMP; and utilization of urea, esculine, glucose, maltose, sucrose, mannitol, and lactose) (Kaźmierczak et al. 2005). The number of strains from particular species among genera corresponded to quantitative occurrence of these bacteria on the skin swabs (Kaźmierczak and Szewczyk 2004). To avoid potential bias, morphological, biochemical features, and susceptibility to antibiotics were considered (Kwaszewska et al. 2009, unpublished data). Cultivation was performed in the following conditions: staphylococci—BHI (Difco) plates supplemented with 5 % sheep blood at 37° for 24 h, corynebacteria—TYT80 medium (Tryptic Soy Agar (Graso), 0.3 % Yeast Extract (Difco) and 0.05 % Tween 80 (Biomedicals INC), and 5 % (*v*/*v*) sheep blood) for 48 h in ambient air (Kaźmierczak and Szewczyk 2004).

### Enzymatic activity

#### Proteolytic activity

Gelatine hydrolysis was conducted on SM-110 medium (Difco) with 10 % gelatine. After 48 h of incubation at 37 °C, culture was covered with saturated solution of diammonium sulfate. Casein utilization was analyzed on P agar with 10 % skimmed milk. For corynebacteria, both media were supplemented with 0.1 % Tween 80 (INC Biomedicals). Proteolytic activity was indicated by the appearance of transparent zone around inoculation streaks.

#### Lipolytic activity

Lipase production was investigated on plates with Triolein (INC Biomedicals) and Rhodamine B (Sigma) according to Kouker and Jaeger (1987) after 48 h of incubation at 37 °C. Orange fluorescence in UV light at 350 nm was considered as positive test for utilization of triolein. Esterase activities were tested on agar plates supplemented with 0.01 % CaCl_2_·H_2_O and 1 % of the following substrates (for each type of esterase respectively): Tween 20 (International Enzymes Ltd.), Tween 40 (Fluka), Tween 60 (Fluka), Tween 80 (INC Biomedicals), and Tween 85 (INC Biomedicals). Determination of lecithinase was performed on egg yolk agar (Gunn 1994). After 48 h of incubation at 37 °C, esterase and lecithinase activities were characterized by a turbid zone around the growth area.

#### Saccharolytic activity

Carbohydrate and sugar alcohols’ (sucrose, mannitol, and glycerol) utilization by staphylococci was tested according to recommendations of International Committee on Systematic of Procaryotes (Freney et al. 1999). Corynebacteria were determined on a liquid medium G-P (according to API-Coryne–BioMérieux) supplemented by inactivated horse serum (3 %) with addition of 1 % of examined substrate (Kaźmierczak et al. 2005). In this case, results were observed for a 2-week incubation period at 37 °C.

#### Urease activity

Standard method was used for staphylococci (Freney et al. 1999), whereas for corynebacteria examination, urea broth (Gunn 1994) was enriched with 0.05 % Tween 80 (INC Biomedicals) and incubation time at 37 °C was 48 h.

### Susceptibility to factors present on the skin

Fresh cultures from solid media were suspended in 0.85 % NaCl standardized to 0.5 McFarland, and 5 μL of each was inoculated onto all types of media (for corynebacteria enriched with 0.1 % Tween 80). To determine the minimal inhibitory concentration (MIC) of palmitic acid, bacterial suspensions were diluted 50 times. Appropriate media without limiting factors were used as strains growth control.

Media used are the following:

- With uric acid: Mueller-Hinton broth (BioMérieux) with uric acid to final concentrations 0.05, 0.067, 0.084, and 0.10 mg/mL.
- With lysozyme: Bacto Pepton (Difco) (1.0 g); yeast extract (Difco) (0.5 %); NaCl (0.5 %); glucose (0.1 %); and Bacto Agar (Difco) (1.5 %) with lysozyme added to concentrations 0.1, 0.2, 0.4, 0. 6, and 0.8 mg/mL.
- To generate osmotic stress: Mueller-Hinton broth (BioMérieux) with the addition of NaCl to receive concentrations (*w*/*v*): 3, 5, 7, and 10 %.
- With low pH: Mueller-Hinton broth (BioMérieux) acidified with 10 % (*v*/*v*) HCl solution up to pH from 3.5 to 6.0 every 0.5.
- With organic acids: Mueller-Hinton broth (BioMérieux) acidified with propionic or lactic acid to receive: pH 5.0, pH 5.5, and pH 6.0.

MIC of palmitic acid (C_16_) was determined on Mueller-Hinton 2 Agar (BioMérieux) plates with palmitic acid (50 g/L predissolved in ethanol) in final concentrations from 0.1 to 4.0 mg/mL every 0.25 mg/mL (Waldon et al. 2002). Bacterial suspensions were spot inoculated (5 μL). The lowest concentration of palmitic acid inhibiting bacterial growth was considered as the MIC value.

### Statistics

Statistical differences were analyzed by applying the Yates’s chi square test. Probability levels of 0.05 or less were considered significant.

## Results

### Substrates for biochemical activity determination

**Fig. 1:**
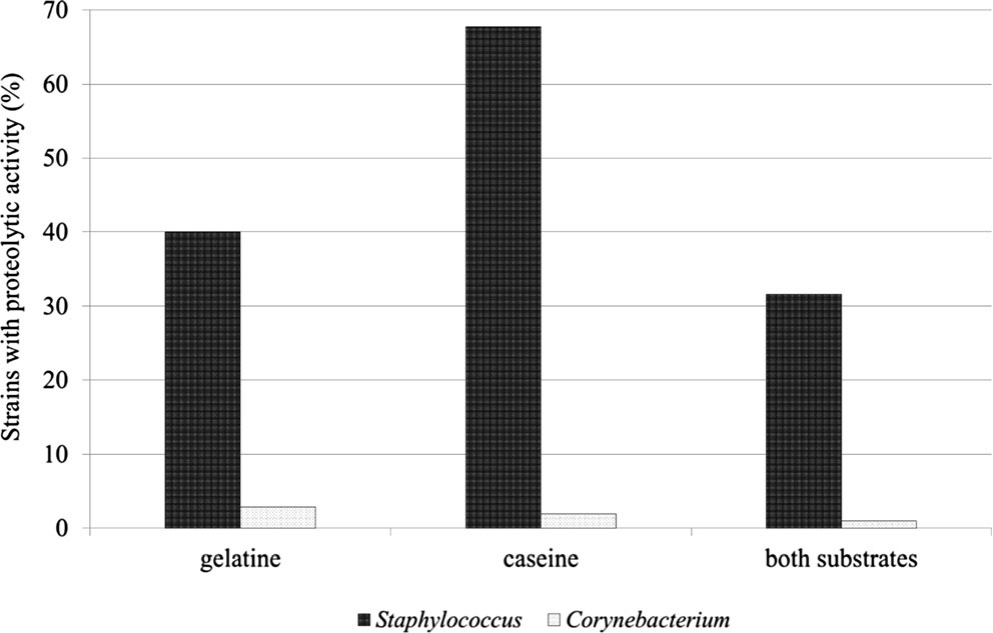
Proteolytic properties of the skin bacteria

Almost all *Staphylococcus* strains utilized proteins, but only few corynebacteria strains had this ability (Fig. 1). Activity of strains belonging to particular species was shown in Table 1.

**Table 1:** Characters of *Corynebacterium* and *Staphylococcus* strains residing on human skin

Genera		*Corynebacterium*					*Staphylococcus*							*p* value^c^
Species		*C. jeikeium*	*C. tuberculostearicum*	*C. afermentans* subsp.* lipophilum*	Other species^a^	All	*S. epidermidis*	*S. capitis* subsp*. capitis*	*S. capitis* subsp*. ureolyticus*	*S. lugdunensis*	*S. haemolyticus*	Other coagulase-negative species^b^	All	
Number of strains (percentage of the representation of the species on the skin)		31 (29.5)	48 (45.7)	10 (9.5)	16 (15.2)	105 (100)	87 (56.1)	25 (16.1)	10 (6.5)	9 (5.8)	8 (5.2)	16 (10.3)	155 (100)	
Number of strains utilizing given substrates (% within species)	Gelatine	0	2 (4.2)	0	1 (6.3)	3 (2.9)	76 (87.4)	8 (32.0)	9 (90.0)	4 (44.4)	1 (12.5)	7 (43.8)	105 (67.7)	0.000
	Caseine	0	1 (2.1)	0	1 (6.3)	2 (1.9)	28 (32.2)	7 (28.0)	7 (70.0)	4 (44.4)	4 (50.0)	12 (75.0)	62 (40.0)	0.000
	Urea	0	0	0	9 (56.3)	9 (8.6)	87 (100)	0	10 (100)	5 (55.6)	0	14 (87.5)	116 (74.8)	0.000
	Sucrose	0	48 (100)	0	6 (37.5)	54 (51.4)	87 (100)	25 (100)	10 (100)	9 (100)	8 (100)	13 (81.3)	152 (98.1)	0.000
	Mannitol	0	0	0	0	0	0	25 (100)	10 (100)	0	8 (100)	6 (37,5)	49 (31,6)	0.000
	Glycerol	0	0	0	0	0	87 (100)	25 (100)	10 (100)	9 (100)	8 (100)	16 (100)	155 (100)	0.000
	Tween 20	29 (93.5)	46 (95.8)	9 (90.0)	14 (87.5)	98 (93.3)	78 (89.7)	4 (16.0)	0	6 (66.7)	5 (62.5)	9 (56.3)	102 (65.8)	0.000
	Tween 40	16 (51.6)	28 (58.3)	3 (30.0)	5 (31.3)	52 (49.5)	48 (55.2)	1 (4.0)	0	2 (22.2)	1 (12.5)	4 (25.0)	56 (36.1)	0.043
	Tween 60	3 (9.7)	0	1 (10.0)	2 (12.5)	6 (5.7)	60 (69.0)	2 (8,0)	1 (10,0)	3 (33,3)	2 (25.0)	5 (31.3)	73 (47.1)	0.000
	Tween 80	24 (77.4)	43 (89.6)	6 (60.0)	9 (56.3)	82 (78.1)	18 (20.7)	0	0	0	0	0	18 (11,6)	0.000
	Tween 85	14 (45.2)	24 (50.0)	5 (50.0)	3 (18.8)	46 (43.8)	67 (77.0)	11 (44.0)	3 (30.0)	3 (33.3)	6 (75.0)	4 (25.0)	94 (60.6)	0.011
	Triolein	31 (100)	48 (100)	10 (100)	16 (100)	105 (100)	82 (94.3)	21 (84.0)	8 (80.0)	9 (100)	7 (87.5)	14 (87.5)	141 (91.0)	0.004
	Lecithine	2 (6.5)	0	0	0	2 (1.9)	36 (41.4)	0	0	3 (33.3)	1 (12.5)	2 (12.5)	42 (27.1)	0.000

^a^ *Corynebacterium pseudogenitalium*, *C. urealyticum, C. accolens, C. diphtheriae* var. *intermedius*, *C. kroppenstedtii*, and *C. macginleyi*

^b^ *S. warneri*, *S. hominis*, *S. caprae*, *S. auricularis*, *S. xylosus*, and *S. simulans*

^c^Statistical significance between *Staphylococcus* and *Corynebacterium* strains analyzed by Yates’s chi square test

Gelatinase activity was expressed mainly by *S. epidermidis* strains. Both substrates (gelatine and casein) were utilized by *S. capitis* subsp. *ureolyticus*. Three strains of *Corynebacterium* spp. utilized gelatine—*C. tuberculostearicum* (two strains) and *C. pseudogenitalium* (one strain). The latter one also utilized casein.

Among the investigated genera, most staphylococci produced urease including all strains of *S. epidermidis* and *S. capitis* subsp. *ureolyticus*. The enzyme was produced only by a few strains of *Coryenbacterium* (9 %) that belonged to *C. urealyticum* and *C. pseudogenitalium* rarely represented on the skin (Table 1).

**Fig. 2:**
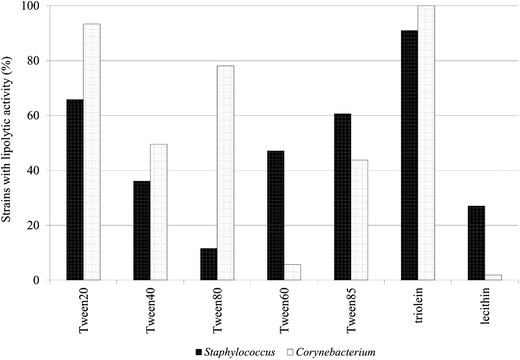
Lipolytic properties of the skin bacteria

Lipolytic properties of strains were evaluated by detection of esterases, lipase, and phospholipase (lecithinase). Esterases were detected on medium containing polyoxyethylene sorbitan monolaurate (Tween 20), monopalmitate (Tween 40), monostearate (Tween 60), monooleate (Tween 80), and trioleate (Tween 85). Esters of laurate, oleate, and palmitate acids were hydrolyzed more frequently by corynebacteria, but more strains of *Staphylococcus* than *Corynebacterium* utilized Tween 60 and Tween 85 (Fig. 2).

Lipase activity with triolein as a substrate was present in the majority of staphylococci and all of corynebacteria. Phospholipase activity was indicated in a greater number of staphylococci (mainly in *S. epidermidis*) than corynebacteria. Only two strains of *C. jeikeium* produced this enzyme (Table 1). *C. jeikeium* and *S. epidermidis* produced lipolytic enzymes of the broadest spectrum of substrates activity. Ten strains of *S. epidermidis* utilized all substrates used in this study.

Apart from being the source of fatty acids, triglycerides are also the source of glycerol. Only staphylococci had the ability to utilize glycerol, and all examined strains expressed this ability. Also, other substrates (sucrose and mannitol) were utilized mainly by staphylococci. Strains of corynebacteria presenting complete lack of saccharolytic activity belonged to *C. jeikeium* and *C. afermentans* subsp. *lipophilum* (Table 1). The same species were not able to utilize proteins. All differences in biochemical activity between *Staphylococcus* and *Corynebacterium* strains were statistically significant.

### Influence of factors present on the skin

**Fig. 3:**
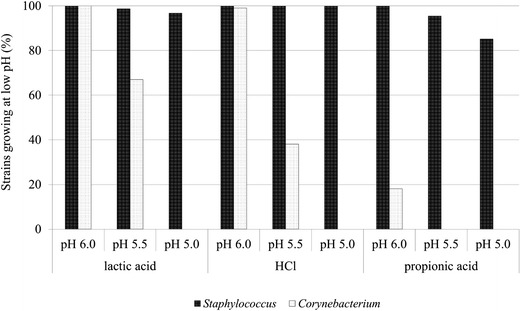
The growth of *Staphylococcus* spp. and *Corynebacterium* spp. at low pH, depending on the type of acid in media

Changes of pH in range from pH 3.5 to 6.0 did not significantly inhibit the growth of staphylococci. Adjustment of pH below 6.0 inhibited the growth of corynebacteria significantly. A type of acid-causing acidification was crucial, as when performed with propionic acid at pH 6.0, the growth of over 80 % of corynebacteria and at pH 5.0 about 15 % of staphylococci were inhibited (Table 2). Corynebacteria most frequently isolated from the skin—*C. jeikeium*, *C. tuberculostearicum*, and *C. macginleyi*—presented the highest tolerance to propionic acid. Acidification by lactic acid caused the reduction of the growth of *Corynebacterium* strains, but *C. jeikeium* multiplied at pH 5.5 (Table 2, Fig. 3). The differences in results in pH below 6 were statistically significant.

**Table 2:** Susceptibility of the skin resident strains to differentiated conditions on the skin

Genera		*Corynebacterium*					*Staphylococcus*							*p* value^c^
Species		*C. jeikeium*	*C. tuberculostearicum*	*C. afermentans* subsp. *lipophilum*	other species^a^	all	*S. epidermidis*	*S. capitis* subsp*. capitis*	*S. capitis* subsp*. ureolyticus*	*S. lugdunensis*	*S. haemolyticus*	Other coagulase-negative species ^b^	all	
Number of strains (percentage of the representation of the species on the skin)		31 (29.5)	48 (45.7)	10 (9.5)	16 (15.2)	105 (100)	87 (56.1)	25 (16.1)	10 (6.5)	9 (5.8)	8 (5.2)	16 (10.3)	155 (100)	
Number of inhibited strains (% within species)	pH 5.0	31 (100)	48 (100)	10 (100)	16 (100)	105 (100)	0	0	0	0	0	0	0	0.000
	pH 5.5	15 (48.4)	29 (60.4)	9 (90.0)	12 (75.0)	65 (61.9)	0	0	0	0	0	0	0	0.000
	pH 6.0	0	1 (2.1)	0	0	1 (1.0)	0	0	0	0	0	0	0	0.844
	Propionic acid pH 5.0	31 (100)	48 (100)	10 (100)	16 (100)	105 (100)	9 (10.3)	5 (20.0)	0	3 (33.3)	3 (37.5)	3 (18.7)	23 (14.8)	0.000
	Propionic acid pH 5.5	31 (100)	48 (100)	10 (100)	16 (100)	105 (100)	3 (3.4)	0	0	1 (11.1)	1 (12.5)	2 (12.5)	7 (4.5)	0.000
	Propionic acid pH 6.0	22 (71.0)	42 (87.5)	10 (100)	12 (75.0)	86 (81.9)	0	0	0	0	0	0	0	0.000
	Lactic acid pH 5.0	31 (100)	48 (100)	10 (100)	16 (100)	105 (100)	3 (3.4)	0	0	0	1 (12.5)	1 (6.3)	5 (3.2)	0.000
	Lactic acid pH 5.5	6 (19.4)	14 (29.2)	5 (50.0)	10 (62.5)	35 (33.3)	1 (1.1)	0	0	0	0	1 (6.3)	2 (1.3)	0.000
	Lactic acid pH 6.0	0	0	0	0	0	0	0	0	0	0	0	0	-
	Uric acid	0	0	0	0	0	0	0	0	0	0	0	0	-
	10 % NaCl	9 (29.0)	10 (20.8)	2 (20.0)	6 (37.5)	27 (25.7)	0	0	0	0	0	0	0	0.000
	Lysozyme	5 (16.1)	4 (8.3)	2 (20.0)	2 (12.5)	13 (12.4)	0	0	0	0	0	0	0	<0.001
Number of strains (% within species)	Palmitic acid MIC < 2 mg/mL (sensitive)	3 (9.7)	21 (43.8)	6 (60.0)	3 (18.8)	33 (31.4)	29 (33.3)	2 (8.0)	0	4 (44.4)	1 (12.5)	4 (25.0)	40 (25.8)	0.396
	Palmitic acid 4 mg/mL ≥ MIC ≥ 2 mg/mL (intermediate)	21 (67.7)	22 (45.8)	3 (30.0)	9 (56.3)	55 (52.4)	44 (50.6)	13 (52.0)	2 (20.0)	5 (55.6)	1 (12.5)	4 (25.0)	69 (44.5)	0.263
	Palmitic acid MIC > 4 mg/mL (resistant)	7 (22.6)	5 (10.4)	1 (10.0)	4 (25.0)	17 (16.2)	14 (16.1)	10 (40.0)	8 (80.0)	0	6 (75.0)	8 (50.0)	46 (29.7)	0.019

^a^ *Corynebacterium pseudogenitalium*, *C. urealyticum*, *C. accolens*, *C. diphtheriae* var. *intermedius*, *C. kroppenstedtii*, and *C. macginleyi*

^b^ *S. warneri*, *S. hominis*, *S. caprae*, *S. auricularis*, *S. xylosus*, and *S. simulans*

^c^Statistical significance between *Staphylococcus* and *Corynebacterium* strains analyzed by Yates’s chi square test

MIC of palmitic acid was determined at range 0.1–4.0 mg/mL. Strains were divided into three groups: sensitive (MIC <2.0 mg/mL), intermediate (4.0 mg/mL ≥ MIC ≥ 2.0 mg/mL), and resistant (MIC >4.0 mg/mL). Most corynebacteria strains were sensitive or intermediate (83.8 %), whereas staphylococci were mainly resistant and intermediate (74.2 %). Difference in the number of resistant strains in both groups was statistically significant (*p* = 0.019) (Table 2). All corynebacteria and staphylococci were resistant to uric acid. All strains of staphylococci were resistant to high salt concentration, whereas 25.7 % of *Corynebacterium* strains were sensitive to high osmotic pressure. Only 12.4 % of corynebacteria were sensitive to lysozyme, and these strains belonged to *C. jeikeium*, *C. tuberculostearicum*, and *C. afermentans* subsp. *lipophilum* species (Table 2).

## Discussion

The skin is the human’s biggest body ecosystem. Although the knowledge of human microbiome is constantly expanding, there are still some areas to discover in this issue (Cogen et al. 2008). Conditions present on the skin enable inhabitation of a quite small variety of bacteria. These are mainly gram-positive bacteria from genus *Staphylococcus*, *Corynebacterium*, *Propionibacterium* as well as *Brevibacterium*, *Dermabacter*, and *Micrococcus*. Their cell wall compositions strengthen resistance to environmental factors. The skin is protected against pathogen colonization by several physiological factors such as low pH, secretion of lipids, lysozyme, and multiple cytokines and skin-associated lymphoid tissue (SALT) (Chiller et al. 2001; Cogen et al. 2008). The phenomenon of colonization resistance is also important because the presence of natural flora protects the skin from being colonized by pathogens (Cogen et al. 2008). The main types of cultured bacteria that form the skin microbiome are coagulase-negative staphylococci and lipophilic corynebacteria and *Propionibacterium* (The Human Microbiome Project Consortium 2012). The knowledge of corynebacteria residing on the skin is particularly limited and even sometimes erroneously discussed in the context of the skin flora with the characteristics of staphylococci. During our experiments, it was proved that lipophilic corynebacteria have different capacity for adaptation on the skin than staphylococci. It was previously discussed that the distribution of representatives of taxons inhabiting the skin depends on the presence of sebaceous and sweat glands in certain regions of the body (Bojar and Holland 2002; Fredricks 2001). Nutrients present on the skin are triglycerides, diglycerides, monoglycerides, glycerol, cholesterol, and its esters, phospholipids and glycoproteins, urea, amino acids, peptides, and uric acid. Exfoliation of the epidermis is the source of protein. Many of these components are products of enzymatic degradation of larger molecules (Bojar and Holland 2002). Our work attempted to determine the association between available nutrients and the bacterial residence capability. The results indicated lack of ability to utilize macromolecular compounds such as proteins or polysaccharides by corynebacteria and simultaneously high efficiency of staphylococci in this respect. It may be assumed that the latter ones provide themselves as well as corynebacteria with the products of their enzymatic activities. It is known that to sustain growth, the skin staphylococci need arginine, cysteine, methionine, valine, and aromatic amino acids which are present on the skin as the products of their proteolytic activities (Bojar and Holland 2002). There is no doubt that it is beneficial for corynebacteria on the principle of cross-feeding as for them, amino acids are also the primary nutrients (Bojar and Holland 2002).

Proteolytic properties of bacteria have a protective role since they inactivate antibacterial proteins from the host organism (Thwaite et al. 2006). Additionally, most bacteriocins secreted by the gram-positive bacteria are hydrolyzed by proteolytic enzymes (Bojar and Holland 2002; Chiller et al. 2001)*.* According to Funke et al. (1997), secretion of proteolytic enzymes by *Propionibacterium* might have a protective effect by providing secure position among the skin flora. The lack of these abilities expressed by the skin corynebacteria weakens their position among the bacterial residents and makes them dependent on other coinhabiting bacteria like staphylococci.

Lipolytic properties of the skin corynebacteria and staphylococci investigated in this paper confirm their act of commensality. Their abilities in this area are clearly complementary and provide the opportunity to benefit from a large variety of compounds. Lipophilic corynebacteria are fatty acid auxotrophs because of the lack of fatty acid synthase (Tauch et al. 2008). In most species, these acids are inserted directly into the cell structures and transformed into corynemycolic acids of the cell wall (Tauch et al. 2005). The coexistence of active lipolytic staphylococci can be helpful for them. Glycerol released after hydrolysis of triacylglycerols cannot be utilized by the lipophilic corynebacteria though it is a good substrate for staphylococci. According to James et al. (2004), this substrate together with lactic, acetic, and propionic acids are the substrates for the formation of volatile fatty acids in nonlipophilic corynebacteria.

The skin inhabitancy capacity was also analyzed in terms of susceptibility to the environmental skin conditions. Secretion of sweat glands generates significant osmotic pressure due to salt present in sweat. Sweat consists of a number of mineral salts—sodium and potassium chloride, calcium, magnesium, and iron salts as well as phosphates—which substantially influence the number of bacterial cells. Over 25 % of investigated *Corynebacterium* strains were sensitive to high concentrations of NaCl in contrast to staphylococci.

Low skin pH inhibits the growth of pathogenic microorganisms, regulates the integrity of the stratum corneum, promotes the activity of β-glucocerebrosidase and acid sphingomyelinase, and inhibits the serine proteinase activity (Behne et al. 2002; Fluhr et al. 2010; Mauro et al. 1998). A number of endogenous mechanisms and metabolism of skin-resident bacteria are responsible for maintaining low pH of the skin (Lambers et al. 2006; Mauro et al. 1998; Tauch et al. 2005, 2008).

It has been frequently reported that the acidity of normal healthy skin varies in the range pH 5–6. Lambers et al. suggested that this value is much lower—approximately 4.7 (Lambers et al. 2006). According to others, a reduction to 4.0–4.5 pH value promotes adhesion of the resident flora to the surface of the skin (Bojar and Holland 2002). In in vitro experiment performed during this study, it was shown that at pH value c.a. 5.5 corynebacteria did not multiply. Moreover, it was indicated that the type of the acid used for establishment of pH was essential. The most noticeable growth of inhibition properties were observed in case of propionic acid; lipophilic corynebacteria growth was inhibited at pH 6.0, and at lower pH, some strains of staphylococci did not multiply. These results are partly contrary to the claims of Ushijima et al. (1984) that propionic acid is one of the factors determining the dominance on the skin not only of its major producer *Propionibacterium* spp. but also coagulase-negative staphylococci.

The skin secretions also contain lysozyme and fatty acids in which 45 mol% is made of saturated ones including palmitic acid (Lieckfeldt et al. 1995). Over 12 % of corynebacteria investigated in this study were sensitive to lysozyme while staphylococci were naturally resistant. Corynebacteria were also more sensitive to palmitic acid than staphylococci. Strains with the activity of esterase capable to hydrolyse palmitic acid from Tween 40 were sensitive to the lowest concentration of this acid used in experiments, which was a phenomenon difficult to explain.

In general, corynebacteria indicated higher sensitivity to environmental factors than staphylococci. It can be compensated by other capabilities of these bacteria such as production of bacteriocins directed against staphylococci (Kwaszewska and Szewczyk 2007).

The existence of microorganisms in a common niche is not only a competition and a constant struggle for their position but also a cooperation and a reciprocal protection which develop their quantitative ratio and must be considered as crucial for the stability of the population on healthy skin.
